# Emergency Laparoscopic Management of Perforative Peritonitis: A Retrospective Study

**DOI:** 10.7759/cureus.20121

**Published:** 2021-12-02

**Authors:** Rafique Umer Harvitkar, Giri Babu Gattupalli, Sakib Najmu, Abhijit Joshi

**Affiliations:** 1 General Surgery, Dr L.H. Hiranandani Hospital, Mumbai, IND; 2 Surgery, Sri Chandra Sekhara Hospital, Hosur, IND; 3 Surgery, Queen Alexandra Hospital, Portsmuth, GBR; 4 Gastrointestinal and Endo-Laparoscopic Surgery, Dr L.H. Hiranandani Hospital, Mumbai, IND

**Keywords:** toxic shock syndrome, malignant hypercapnia, pneumoperitoneum, laparoscopy, peritonitis

## Abstract

Background

Peritonitis was previously considered a contraindication for minimally invasive surgery due to the risk of malignant hypercapnia partial pressure of carbon-dioxide (PCO2) and toxic shock syndrome. The objective of this retrospective study was to evaluate the role of laparoscopic surgery (LS) in selected patients with perforative peritonitis and to study its feasibility, safety, and outcomes.

Patients and methods

This was a retrospective study of 25 patients spanning over five years from 2015 to 2020. This study comprised all patients who were diagnosed with perforative peritonitis on preoperative physical/clinical examination, radiological evaluations, and who were stable enough to withstand pneumoperitoneum. Patients were evaluated for causes, operative time, duration of hospital stay, intra-, and postoperative complications, time taken to resume normal activity, and conversion to open surgery. Data was extracted from the hospital electronic medical records, for the above-mentioned parameters.

Results

Twenty-five patients with perforative peritonitis underwent diagnostic and therapeutic LS in our institute. The mean age was 46 years (35-79 years). Ten patients (40%) were diagnosed with gastro-duodenal perforation. Out of these ten patients, ninepatients (90%) were managed totally laparoscopically, while one patient (10%) required conversion to open surgery. There were 15 patients (60%) with small bowel perforation. Thirteen of the 15 patients were managed laparoscopically, with the remaining two requiring conversion to open surgery. The average time taken for the procedure was 90 minutes. The mean time to initiate the postoperative peroral liquid diet was 3.4 days. The mean postoperative stay was 6.9 days. The time taken to resume normal activity was 10-12 days.

Conclusions

Laparoscopic management is feasible and safe for patients with perforative peritonitis. Careful patient selection and the surgeon’s experience with the procedure are critical determinants of success.

## Introduction

Perforative peritonitis is one of the most common causes of abdominal surgical emergency and intervention. A meticulous surgical technique with systematic evaluation of the peritoneal cavity, complete evacuation of purulent fluid, optimum closure technique, and thorough peritoneal toilet are mandatory for good outcomes.

Historically, peritonitis was considered an absolute or relative contraindication for laparoscopic surgery (LS) due to multiple factors and arguments [1,2]. Firstly, the theoretical risk of hypercapnia due to increased absorption of carbon dioxide is directly related to increased intraabdominal pressure (IAP), infection, and inflammation. Secondly, the risk of toxic shock syndrome due to increased IAP results in the passage of toxins and bacteria into the general circulation. Lastly, the surgeons opted not to use laparoscopic therapy for perforative peritonitis due to inflamed and friable bowel, limited working space, and difficulty manipulating the bowel [3,4].

However, greater acceptance of laparoscopy in recent years has encouraged surgeons to use it due to its proven benefits of less pain, short hospital stays, faster recuperation, and decreased morbidity [4-6]. Performing diagnostic laparoscopy in cases of suspected viscous perforation or peritonitis has the advantage of identifying an occasionally unexpected pathology. If favorable abdominal pathology is discovered, it can be managed and repaired laparoscopically. However, if laparoscopy-assisted conversion is to be conducted, it has the advantage of a more selective and shorter laparotomy incision. According to the European Association for Endoscopic Surgery (EAES) guidelines, in cases of the peritonitic abdomen, laparoscopy is no longer an absolute contraindication [7,8,9].

Encouraged by multiple studies and their findings, we extended the many benefits of LS to patients with localized or generalized peritonitis and conducted this retrospective study to analyze the outcomes.

## Materials and methods

We performed a retrospective review of patients diagnosed with peritonitis, who underwent LS at our institution, from 2015 to 2020. Our study included all patients who were diagnosed with perforative peritonitis based on imaging investigations. Intravenous fluid resuscitation and the prevention of secondary organ dysfunction were of the utmost importance for treating these patients. Empirical broad-spectrum systemic antibiotic therapy was initiated at admission in line with the hospital’s antibiotic policy (injection ceftriaxone 1 gm intravenous and then BID for 5-7 days, injection metronidazole 500 mg intravenous and then TID for five days), and the further course was tailored according to the antibiotic culture and sensitivity report of the infected peritoneal fluid. Nasogastric tube for decompression, per urethral catheter, and adequate analgesia was instituted immediately on admission. Before the surgical intervention, dyselectrolytemia and coagulation abnormalities, if present, were corrected to the maximum extent possible.

**Figure 1:**
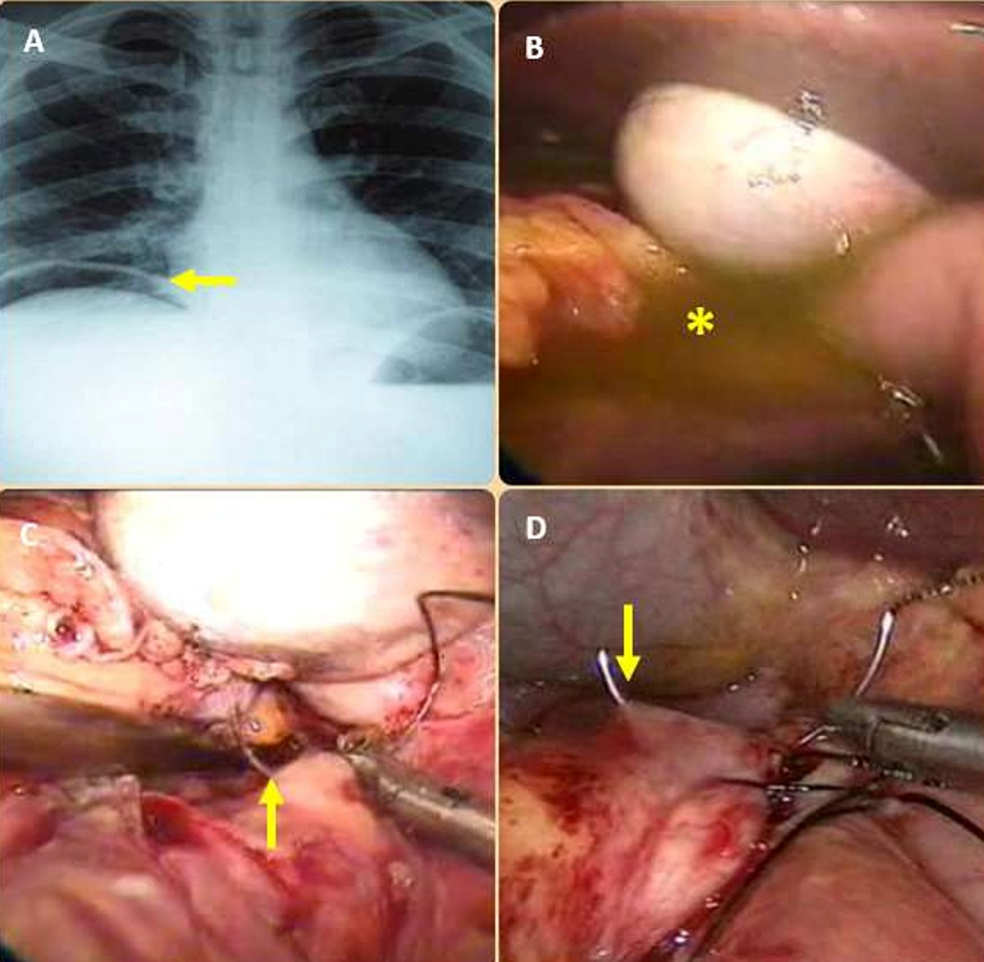
A) shows an X-ray chest with both domes with classical free gas under the right dome of diaphragm (yellow arrow), indicating a gastrointestinal perforation B) shows typical bilious peritoneal contamination of an upper GI perforation (yellow asterisk C) shows suture closure of the D1 perforation underway D) suture closure pre pyloric perforation

In our institute, only those patients of perforative peritonitis who satisfy the following criteria are subjected to LS: all patients with hollow visceral perforation, as confirmed by a plain X-ray chest with domes showing free gas under the right diaphragm dome (Figure 1A) or plain computed tomography of the abdomen revealing free gas in the peritoneal cavity; patients who present with peritonitis early, i.e., within the first 24 hours of the onset of acute symptoms.

Our exclusion criteria were: hemodynamically compromised and unstable patients (blood pressure less than 90/60 mmHg, pulse more than 110 beats per minute); patients with irreversible coagulopathy or hypercapnia greater than 50 torrs; patients with previous extensive open abdominal surgeries (two or more); patients with compromised cardiovascular or respiratory systems; patients with known cases of underlying conditions, such as Crohn’s disease, ulcerative colitis, diverticulosis and diverticulitis.

Data for the following parameters were extracted from the hospital's electronic medical records: sex, age, surgical result (time/procedure/result), the reason for the conversion (where applicable), etiology of the perforation, perioperative and postoperative complications, duration of hospital stay, and mobility. We monitored the patients for two weeks after surgery.

Procedure

Preoperative Preparation

Preoperative preparation is in the form of adequate intravenous fluid replenishment, antibiotic initiation, and appropriate antithrombotic prophylaxis. Nasogastric decompression should always be conducted in each patient. It facilitates decompression of the stomach and the rest of the proximal bowel, which is usually in the ileus due to peritoneal contamination. This minimizes the risk of aspiration while under anesthesia, and deflation of the distended bowel enables relatively easier and safer handling during surgery.

Patient Position and Surgical Technique

The patient is placed in a supine position with legs straight and split. He/she is firmly strapped-fixed to the table at the lower chest level, allowing for steep Trendelenburg, reverse Trendelenburg, and right and left lateral positions. The pressure points and contact areas are adequately padded. In patients without scars from previous surgery on the abdomen, the umbilical area is our preferred point of entry (supra or infra umbilical, depending on the adequacy of the umbilicus to pubis distance). If the bowels are extremely distended and the abdomen too tense, we prefer to perform a direct 10-mm blunt trocar insertion by the open technique. In other situations, where the nasogastric tube has aspirated copious amounts and the abdomen is relatively soft, we prefer to institute pneumoperitoneum through the targeted site using conventional Verress’s needle technique.

In patients who have scars from previous abdominal surgery, we prefer to institute pneumoperitoneum through the Verres needle at Palmer’s point (a relatively safe point for entry, on the left midclavicular line two finger breadths below the costal margin). Then, we insert a 5-mm trocar at the same point and a peripheral “bird’s eye view” of the abdomen was obtained using a 5-mm telescope inserted through this trocar. Central trocars are then inserted, carefully avoiding any adhesions (if present), under the vision provided by this 5-mm telescope. Dense adhesions, if present, are first lysed through additional peripheral trocars inserted in “safe areas,” before inserting the central trocars. After inserting the central 10-mm trocar, we switched to a 10-mm telescope. The operating surgeon stands in between the patient’s legs while the camera assistant surgeon stands on the patient’s right side and the second assistant surgeon stands on the patient’s left side. Then, we insert the right- and left-hand 5-mm working trocars on either side of the umbilical area 10-mm optic trocar. We then examine the upper abdomen and the gastro-duodenal area for obvious perforations. Also, a careful inspection of the nature of the peritoneal contaminating fluid provides a reliable clue as to the site of perforation.

**Figure 2:**
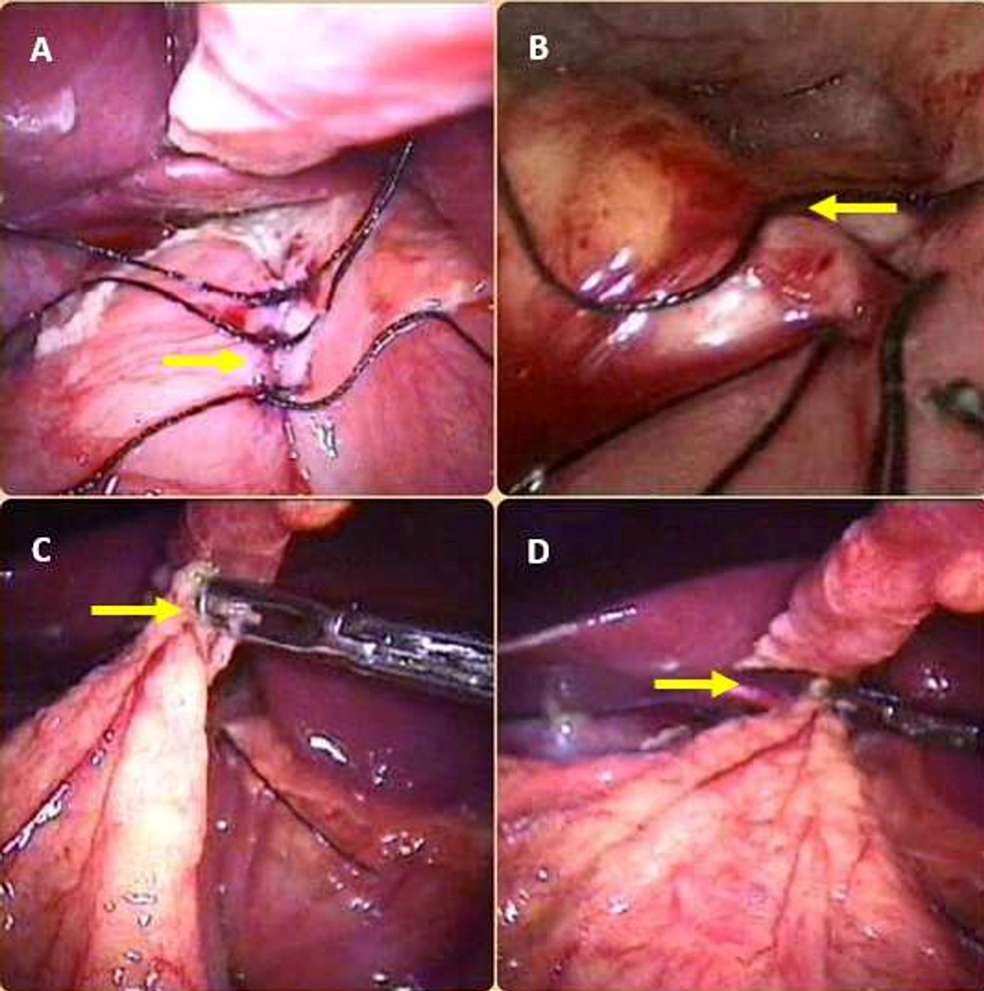
A) shows completed suture line with ends of suture material kept long so as to accommodate the omental patch B) completed suture line C, D) show an omental onlay patch being placed on the suture line and secured in place by tying the long ends of the suture material

**Figure 3:**
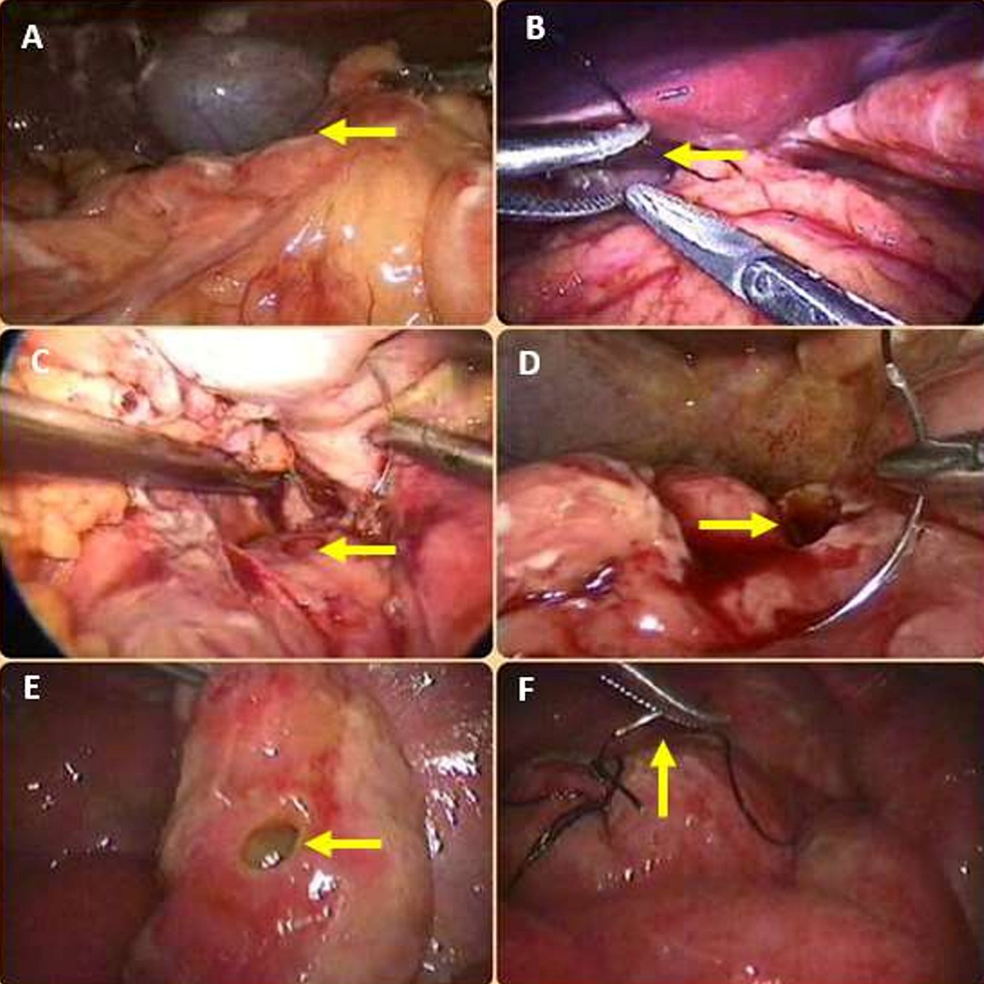
A) shows the omental onlay patch being readied; B) shows an omental onlay patch being placed on the suture line and secured in place by tying the long ends of the suture material; C) shows an anterior wall of D1 perforation (yellow arrow); D) shows a pre-pyloric perforation; E) shows a typhoid terminal ileal perforation (yellow arrow); F) shows its suture closure underway (yellow arrow)

A bilious or non-bilious contamination fluid with or without food particles but without feculent odor (Figure 1B) indicates upper gastrointestinal perforation. In these cases, an additional 5-mm trocar is inserted in the left lateral abdomen. Through this, the assistant’s atraumatic grasper is inserted. This instrument grasps the anterior wall of the stomach and retracts it laterally to adequately expose the pre-pyloric or duodenal perforation. The gastro-duodenal perforations were sutured closed using the two working trocars with 2-0 or 3-0 silk, with simple interrupted sutures (Figures 1C, 1D). The individual stitch ends were kept long (Figures 2A, 2B). After optimum suture closure of the peptic perforation, an omental patch was mobilized and placed over the suture line (Figures 2C, 2D, 3A). The long ends of the sutures were then tied around this omental patch, to maintain its position (Figure 3B). Before attempting suture closure in suspected malignant gastric perforations, we performed an edge biopsy.

If the sutures cut through the indurated suspected malignant tissue, or otherwise through the edges of a perforated chronic peptic ulcer, just an onlay omental patch is fixed in place without prior suture closure of the perforation, with sutures taken farther away from the edges of the perforation. After sucking out all the peritoneal contamination, a thorough peritoneal toilet was administered with normal saline. This is where laparoscopy can be inferior to open surgery on two counts: (i) thick pus and pus flakes are difficult to suck out into a 5-mm suction cannula, and (ii) the optimum visualization of all the recesses, nooks, and corners of the peritoneal cavity is not always guaranteed. Hence, there is a genuine risk of missing collections and formation of postoperative intraperitoneal abscesses. The first issue, thick pus and pus flakes not getting sucked into a 5-mm suction cannula are solved by careful and judicious use of a 10-mm suction cannula. A 10-mm cannula sucks out the difficult pus flakes but poses a real risk of causing “suction injury” to the vulnerable bowel. We avoided this by utilizing the 10-mm cannula only when its tip is visible and in relatively open spaces. To solve the second issue, our unit has implemented the “as many trocars as it takes” policy. This means that we will never hesitate to insert 1-2-3 additional trocars at appropriate places to retract dilated bowel obstructing the view, suck out residual collections, and provide a toilet; even after the main phase of the surgery - the suture closure of the perforation is completed. This enabled us to provide significant and comprehensive peritoneal toilets to all of our cases, and we have not had a single postoperative infected intraperitoneal collection in any of our patients in this series. At the end of the operation, a 32 Fr tube drain was inserted through the right lateral working trocar site, placed in situ in Morrison’s pouch, and suture-fixed to the skin. For patients with massive peritoneal contamination, we inserted an additional drain in the pelvis through the left-hand working trocar site.

When the peritoneal contamination is feculent (color and/or odor), we suspected a lower gastrointestinal, i.e., small bowel perforation. We inserted the suprapubic 10-mm trocar after evaluating and ruling out an upper gastrointestinal perforation, which then became our primary optic trocar. An additional 5-mm trocar was inserted in the right iliac fossa (RIF). Once the telescope is shifted to the suprapubic trocar, RIF and umbilical trocars become the surgeon’s left and right-hand working ports, respectively. A systematic “bowel walk” was now initiated, starting from the ileocecal junction to the duodenojejunal flexure. We believe that the suprapubic optic trocar provides an optimum view of the central abdomen, thus enabling accurate identification and localization of the pathology and eventual therapy. We believe that while dealing with dilated bowels with a suboptimal view, one should not hesitate to insert an extra trocar or two at optimum locations to enable insertion of extra instruments, such as the fan retractor, for better atraumatic retraction of dilated bowel and safer surgery.

Once the small bowel perforation had been located, we conducted a careful inspection to determine the size of the perforation and any accompanying findings, such as a stricture, adhesive band, etc. In such scenarios, segmental resection and stapled cum sutured end-to-end small bowel anastomoses are preferable. In standalone perforations, the edge was freshened by excising a thin sliver of tissue circumferentially along the edge of the perforation. The same specimen was also sent for histopathological examination. Then, the perforation was suture closed using 3-0 silk in two layers, using inner continuous and outer simple interrupted sutures. An omental wrap was performed around the suture line if the bowel is extremely inflamed and friable. The remaining steps of peritoneal toilet and drainage tube(s) insertion remain the same. However, only the site of the right drain differs. It was placed near the anastomosis.

## Results

This study comprised 25 individuals who had perforative peritonitis and underwent LS up until March 2020. There were 15 males (60%) and 10 females (40%) with a mean age of 46 years (35-79 years). Ten patients (28%) were diagnosed with gastric and duodenal perforations. Out of these 10 patients, nine (90%) were managed laparoscopically, while one patient (10%) required conversion to open surgery due to massive peritoneal contamination and unclear anatomy. Peptic ulcer disease was the etiology of all our gastro-duodenal perforations. All 10 patients with peptic gastro-duodenal perforations gave a history of taking intermittent empirical treatment with proton pump inhibitors (PPI) for acute peptic gastritis, on the advice of the medical gastroenterologist. However, none of them were actively on PPI therapy just prior to the occurrence of the surgical emergency. Out of the 10 patients with gastro-duodenal perforations, nine had duodenal (anterior wall of the first part of the duodenum-D1) perforations (Figure 3C), while one had a pre-pyloric perforation anteriorly on the stomach wall (Figure 3D). We performed an edge biopsy on this one patient before suture closure. There were 15 patients (60%) with small bowel-ileal perforations. Out of these 15 patients, 13 (87%) were managed laparoscopically, while two (13%) required conversions due to unclear anatomy and technical difficulty. Out of the two patients who were converted to a laparotomy, one had a tuberculous stricture-perforation complex and could not be managed laparoscopically due to technical difficulty caused by the surrounding massively dilated small bowel.

The second converted patient had an isolated terminal ileal perforation due to acute abdominal trauma caused by a road traffic accident. This patient also had a large small bowel mesenteric tear that caused hemoperitoneum, which compromised optimum visualization. Seven of the 13 patients with small bowel perforation managed successfully by laparoscopy underwent suturing of isolated small perforations (Figures 3E, 3F), while six required segmental resections with end-to-end small bowel anastomosis. Histopathology revealed that all seven of these patients had intestinal (typhoid) perforations. All the six patients who needed a small bowel resection anastomosis had a stricture-perforation complex. Histopathology revealed that all of these were tuberculous in origin. In the six patients who required a small bowel resection anastomosis, the same was performed totally laparoscopically using blue cartridges loaded on an Endo GIA (Medtronic, Dublin, Ireland) surgical stapler.

We divided the mesentery using a harmonic scalpel. At the end of the anastomosis, the mesenteric defect was suture closed. A systematic and thorough “bowel walk” was also performed in these six patients to identify/rule out additional concurrent strictures, both proximal and distal to the perforation. Furthermore, the ileocaecal junction was visualized and palpated with a “soft” atraumatic bowel grasper to identify concurrent ileocaecal tuberculosis. None of these six patients had any concurrent additional small bowel strictures or ileocecal tuberculosis. In our study, only three patients (12%) required conversion, while the other 22 cases (88%) were effectively managed laparoscopically. The operation took an average of 90 minutes to complete. The mean time to start an oral liquid diet was three to four days. The mean postoperative stay was 6.9 days. The time taken to resume normal activity was 10-12 days. Mild complications were observed in four (16%) patients during the immediate postoperative period and two weeks after surgery. Three patients were treated conservatively for minor trocar site wound infections (two with grade IIA and one with grade IIB according to the Southampton wound grading system). All these three patients were cases of solitary typhoid perforations of the terminal ileum. The remaining patient experienced mild paralytic ileus, which got resolved post electrolyte supplementation. None of the patients in this series had a postoperative leak through the suture/staple line. None of the 14 patients (one pre-pyloric ulcer perforation and 13 small bowel perforations) who underwent a histopathological study of their operative specimens, had a malignancy.

Table 1 shows the patient demographics, perioperative data, and etiological information of all the patients of this series.

**Table 1:** The patient demographics, peri-operative data, and etiological information

Characteristics	Number (n = %)
Total Number of cases	25
Conversion to open	03(12%)
Completed by Laparoscopy	22(88%)
Mean Duration of Surgery (Minutes)	90
Meantime to Liquid Feed (Days)	3.4 days
Mean Stay	6.9 Days
Causes: a) Peptic Perforations	10(40%)
b) Enteric Perforations	07(28%)
c)Tuberculous Perforations	07(28%)
d)Trauma	01(4%)

## Discussion

Open surgery, i.e., laparotomy, has conventionally been the standard of therapy for patients with perforative peritonitis all over the world. The feasibility of laparoscopy in an acute abdomen is reported to be approximately 90%, but as high as 98% in other cases, as reported by Kirshtein [10]. In the management of abdominal emergencies, there is no difference for absolute and relative contraindications as far as both laparoscopy or open procedures are concerned [11-12]. However, for peritonitis, there is a concern that pneumoperitoneum (increased CO2) may enhance bacteremia and endotoxemia due to increased IAP [7,13,14]. C.A. Jacobi et al. concluded that the inflammatory response was significantly higher in laparotomy in their study to investigate the influence of laparotomy and laparoscopy on local and systemic inflammation [15]. Acute phase reaction markers, e.g., ceruloplasmin, C-reactive proteins (CRP), fibrinogen, haptoglobin, serum lactate, were lower in laparoscopy than in laparotomy. Over the last few years, there has been an increase in the number of studies supporting laparoscopy for peritonitis. We also agree with the EAES clinical guidelines in favor of LS and creating pneumoperitoneum in the peritonitic abdomen [7]. The diagnostic accuracy of laparoscopy was 100% in our study, compared to international literature reported to be 89%-100% [13].

Laparoscopic Surgery's high and specific diagnostic yield is critical, especially in patients with suspected gastrointestinal perforation (peritonitis), since it allows for a better and thorough examination of the peritoneal cavity and detection of concomitant diseases. In cases of unclear preoperative diagnosis, laparoscopy can shorten the observation period and avoid the need for exorbitant haematological and radiological investigations [14,15].

Laparoscopic allows us to perform the same surgical procedure as open surgery. Many patients with peritonitis do not have an evident perforation, but rather an inflammatory necrotic zone (e.g., edema/abscess) formation. Such patients can be safely treated with peritoneal lavage and broad-spectrum antibiotic therapy. This may allow us to arrange for a second LS for the underlying pathology if necessary, such as in cases of sigmoid resection with diverticular patients under elective conditions [16,17]. A surgeon should never contemplate conversion to open surgery as a defeat. Instead, he/she should constantly keep in mind that by adopting the LS method, he/she can select the most appropriate incision for the patient if the decision to convert to open surgery is made. The results of our study indicated the compatibility and feasibility of LS in the management of selected cases of peritonitis.

The complications can undoubtedly reduce with careful patient selection, increased skill, and confidence with the surgical technique. Although the exact economic benefits of LS are difficult to quantify, it significantly reduces wound infection rates. More importantly, it completely negates the possibility of major wound complications, such as incisional hernias and burst abdomen, both of which would require additional surgical correction, thereby increasing patient suffering and the financial burden on the healthcare infrastructure. Therefore, we believe that the total prevention/avoidance of major wound complications is the most significant advantage of laparoscopic management of selected cases of perforative peritonitis. Also, it leads to faster recovery and return to work [18]. Lastly, a small high-pressure operating room with well-trained and experienced surgeons working with a well-trained team is necessary for the procedure’s success. In our study, all the procedures were performed by a single surgeon with extensive experience and expertise in advanced laparoscopic surgeries.

Although our results are favorable toward laparoscopy, we do not think that they are comparable to a group of patients for whom we would prefer open surgery due to more severe symptoms (septic shock, hemodynamic instability, cardiopulmonary impairment, etc). Our study is also limited by its small size and retrospective nature. Despite these limitations, we believe that the results obtained are robust enough to indicate the advantages of laparoscopic therapy for gastrointestinal perforative peritonitis.

## Conclusions

As seen in this study, laparoscopic surgery has a definite role in the surgical management of selected cases of perforative peritonitis. Evaluation of the results of this study shows that it is feasible, safe and has positive outcomes for the patient. Careful patient selection, meticulous surgical technique, and the surgeon’s experience with the procedures enable a high success rate. As demonstrated in this case series, logical and predefined inclusion/exclusion criteria coupled with enhanced laparoscopic surgical skills provide the benefits of minimal access surgery to carefully selected patients with bowel perforations.
