# Correction: Emergency Laparoscopic Management of Perforative Peritonitis: A Retrospective Study 

**DOI:** 10.7759/cureus.c58

**Published:** 2022-03-10

**Authors:** Rafique Umer Harvitkar, Giri Babu Gattupalli, Sakib Najmu, Abhijit Joshi

**Affiliations:** 1 General Surgery, Dr L.H. Hiranandani Hospital, Mumbai, IND; 2 Surgery, Sri Chandra Sekhara Hospital, Hosur, IND; 3 Surgery, Queen Alexandra Hospital, Portsmuth, GBR; 4 Gastrointestinal and Endo-Laparoscopic Surgery, Dr L.H. Hiranandani Hospital, Mumbai, IND

The following typos were present in the original published version of this article and have now been corrected:

1. Materials & Methods, first paragraph after Figure 1, first sentence: pulse changed from 10 bpm to 110 bpm
2. Materials & Methods, first paragraph after Figure 1, first sentence: the following clause was removed: "patients unable to withstand pneumoperitoneum remove this point"

Cureus regrets that these errors were not identified prior to publication.

